# Converting tumors into vaccine manufacturing factories: DC recruitment, activation and clinical responses with a flt3L-primed in situ vaccine for low-grade lymphoma [nct01976585]

**DOI:** 10.1186/2051-1426-2-S3-P45

**Published:** 2014-11-06

**Authors:** Nina Bhardwaj, Miriam Merad, Seunghee Kim-Schulze, Beth Crowley, Thomas Davis, Tibor Keler, Andres Salazar, Joshua Brody

**Affiliations:** 1 Icahn School of Medicine at Mt Sinai, New York, NY, USA; 2 Mount Sinai School of Medicine, USA; 3 Celldex Therapeutics, Inc., USA; 4 Oncovir Inc, USA

## Background

Lymphomas are the 5^th ^most common cancer in the U.S. and the most prevalent amongst these, low-grade B cell lymphomas are incurable with standard therapy. Previously, we completed three trials combining low-dose radiotherapy (XRT) with intratumoral administration of a TLR9 agonist (CpG) for patients with low-grade lymphoma, an approach we refer to as '*in situ *vaccination'. We demonstrated induction of anti-tumor CD8 T cell responses as well as clinical remissions of patients' **non**-irradiated sites of disease, lasting up to 4+ years. Not all patients mounted CD8 T cell responses, possibly due to the paucity of intratumoral dendritic cells (DC), given the exceptional ability of these cells to endocytose dying (e.g. irradiated) tumor cells for cross-presentation to anti-tumor CD8 T cells. Increasing intratumoral DC may improve the *in situ *vaccine.

## Methods

**Figure 1:**
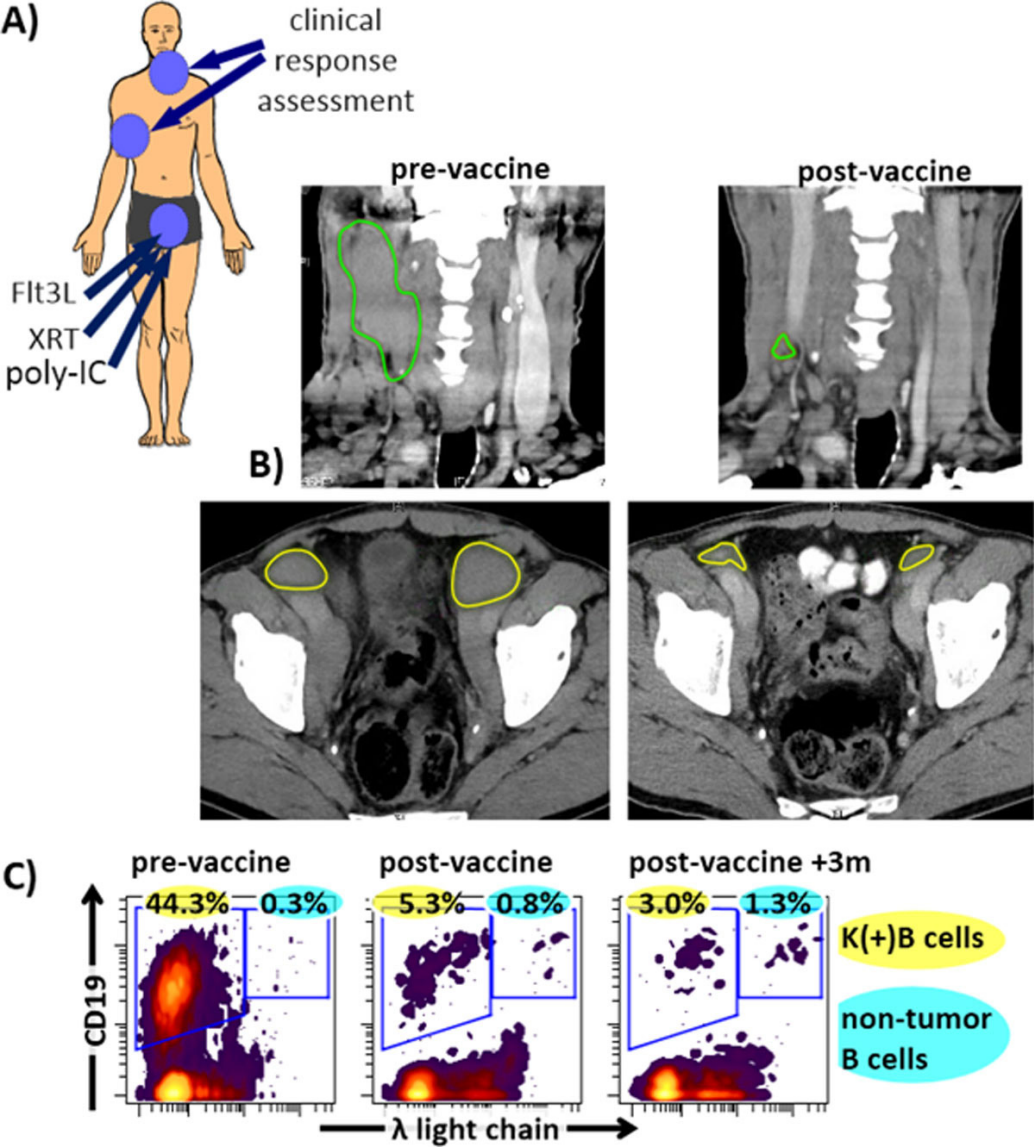
**Flt3L-primed insitu vaccine**. A) Schema B) Regression of bulky (untreated) cervical and external iliac adenopathy and C) Decrease in peripheral blood lymphoma (included in CD19+λ-) cells with concurrent increase in non-tumor (CD+λ+) B cells.

**Flt3L**, the predominant DC differentiation factor, induces tumor leukocyte infiltration and regression of lymphoma pre-clinically, and a formulation of this cytokine, CDX-301, mobilizes BDCA-1 and BDCA-3 DC subsets as seen in a recently completed Phase I trial. These DC subsets respond to several TLR agonists and cross-present antigens more effectively than plasmacytoid DC. We have initiated a Phase I/II study of a new iteration of the *in situ *vaccine, adding Flt3L-priming and replacing the prior TLR9 agonist with the TLR3 agonist poly-ICLC (Figure 1A). The vaccine consists of:

Intratumoral Flt3L administration to increase DC within the tumor

Low-dose XRT to induce immunogenic tumor cell death and release tumor-associated antigens

Intratumoral poly-ICLC administration to *activate *tumor antigen-loaded DC

## Results

Six patients have been enrolled, and two patients have completed therapy. Treated patients have shown marked increase in both BDCA1 and BDCA3 intratumoral DC after treatment with Flt3L as well as DC activation after treatment with XRT and poly-ICLC. Both treated patients have demonstrated partial remissions of *untreated *sites per International Working Group criteria, persisting or improving for >4 months after vaccination. These include regressions of bulky lymph nodes (Figure 1B), as well as peripheral blood (Figure 1C) and bone marrow disease. In one patient with significant peripheral blood tumor burden we observed >10-fold decrease in malignant B cells with concurrent increase in non-tumor B cells, suggesting some degree of cell specificity in the tumor-killing mechanism. Adverse effects observed to date have been mild.

## Conclusions

Preliminary results suggest that the Flt3L-primed *in situ *vaccine is feasible, safe and immunologically and clinically effective. The study is ongoing.

